# Global Bias‐Aware Synthesis of Meta‐Analyses Reveals Agroforestry's Potential for Improving Soil Health

**DOI:** 10.1111/gcb.70960

**Published:** 2026-06-18

**Authors:** Rubeaud Camille Manon, Beillouin Damien, Köthke Margret, Six Johan, Walder Florian, Schievano Andrea, Kay Sonja

**Affiliations:** ^1^ Agroscope Zürich Switzerland; ^2^ Sustainable Agroecosystems Group, Department of Environmental Systems Science Swiss Federal Institute of Technology, ETH Zürich Zurich Switzerland; ^3^ CIRAD, UPR HortSys, Univ Montpellier Montpellier France; ^4^ Thünen Institute of Forestry Hamburg Germany; ^5^ Joint Research Centre European Commission Ispra Italy

**Keywords:** erosion, nutrient leaching, robust Bayesian meta‐analysis, soil biological quality, soil chemical quality, soil functions, soil organic carbon, soil physical quality, water regulation

## Abstract

Agroforestry systems (AFS), which integrate trees into agricultural landscapes, offer a promising strategy for climate change mitigation and biodiversity restoration. The trees can access additional resources, improving soil functions and contributing to soil regeneration through increased organic matter inputs. However, evidence is scattered and thus hindering unified global quantification of impacts. Here, we present the first bias‐adjusted global quantitative synthesis of agroforestry effects on distinct soil and water attributes, drawing on 1590 primary studies summarized in 26 meta‐analyses. We evaluated each meta‐analysis against 16 standardized quality criteria. Less than half of the meta‐analyses met > 75% of these criteria, while most did not weigh effect‐sizes or assess heterogeneity and publication bias. We quantified primary studies overlap, finding an overall redundancy of 18% across all soil parameters, most of which arise from studies reporting on soil carbon. Accounting for meta‐analysis quality and bias, AFS tend to increase soil organic carbon by 20%, improve chemical soil quality by 59%, physical soil quality by 22%, and enhance water regulation by 71%. Biological soil quality (86%), nutrient leaching reduction (67%), and erosion control (6%) also benefit from AFS, though with higher heterogeneity. Evidence is extensive for soil organic carbon and chemical properties (73% of studies), whereas it remains limited for AFS effects on biological (< 12%), physical (< 19%), and water‐regulation traits (< 27%). 58% of meta‐analyses report no details at all on system characteristics, such as stand age, species diversity, or tree density, limiting the identification of AFS management practices that maximize soil health attributes and highlight the need for standardized definitions of agroforestry and consistent sampling protocols. By systematically evaluating meta‐analysis quality and study overlap, this synthesis provides a robust framework to distinguish reliable from uncertain outcomes and demonstrates the capacity of agroforestry to deliver multifunctional benefits, particularly regarding soil health.

## Graphical abstract

This second‐order meta‐analysis synthesizes evidence across diverse agroforestry systems (silvoarable, silvopastoral, tree‐landscape features, and perennial shaded systems) to assess their effects on multiple soil health attributes, including soil organic carbon, biological, chemical, and physical properties, water regulation, nutrient leaching, and erosion control, across arid, mediterranean, temperate, and tropical climates. Moving beyond the simple aggregation of existing meta‐analyses, this study explicitly addresses primary study overlap, quality‐weighted effect sizes, and bias‐ and heterogeneity‐adjusted estimates, enabling the identification of reliable impacts, uncertain outcomes, and critical research gaps by soil health attribute, system type, and climate.

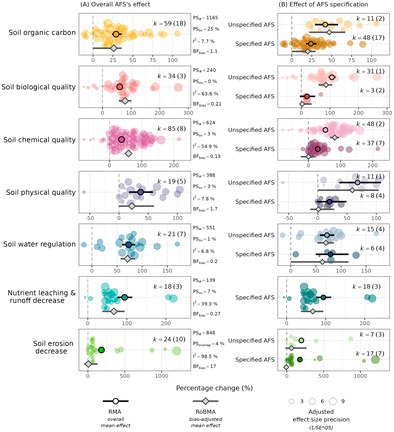

## 1 Introduction

Agroforestry, the integration of trees into agricultural systems, is considered a nature‐based solution for both adapting to and mitigating climate change, while simultaneously enhancing biodiversity in cultivated landscapes (Azhar et al. 2024; Kay et al. 2019). Beyond providing aboveground habitat for a wide range of plant and animal species, trees also support key belowground processes, including carbon, nutrient, and water cycling (Cardinael et al. 2020). As these processes underpin critical soil functions, often summarized as *soil health*, assessing the impact of agroforestry systems (AFS) on soil health attributes is essential for evaluating whether, and to what extent, AFS contributes to the resilience of agroecosystems (Dollinger and Jose 2018; Van Noordwijk et al. 2019).

In this study, we use the term *soil health* as a comprehensive framework encompassing biological, chemical, and physical soil properties that reflect the soil's capacity to sustain key ecosystem functions and services (Bünemann et al. 2018; Harris et al. 2022; Lehmann et al. 2020). Although *soil health* and *soil quality* are often used interchangeably in the literature (Bünemann et al. 2018), the concept remains contested regarding its definition and measurability (Baveye 2021; Janzen et al. 2021). Our study contributes to a system‐level understanding of the impact of AFS on soil systems, without supposing that the term *soil health* can be unambiguously defined.

In an agricultural context, healthy soils are characterized by high biological activity, the presence of available nutrients, stable structure, efficient water retention, maintenance of water quality, and resilience against degradation processes such as erosion (Lehmann et al. 2020). AFS can enhance these attributes through the ability of trees to access above‐ and below‐ground resources, such as water, light, and nutrients that are often inaccessible to crops, thereby increasing overall biomass production (Cannell et al. 1996; Niether et al. 2020).

Additional organic matter input from leaf litter, root biomass, and rhizodeposition can elevate soil organic carbon stocks (Beillouin et al. 2023; Cardinael et al. 2018; Chatterjee et al. 2018) and stimulate root–microbe interactions, including mycorrhizal networks (Battie‐Laclau et al. 2020; de Carvalho et al. 2010; Dierks et al. 2021). This can lead to a marked increase in the diversity and abundance of soil microbial communities, particularly in the vicinity of trees (Guillot et al. 2021; Marsden et al. 2020). It has been shown that tree rows in silvoarable systems further foster soil macrofauna like earthworms by providing a refugium (Cardinael et al. 2019). Biopores formation by tree roots and increased macrofauna, associated with the additional soil organic carbon, are supposed to improve soil structure, aggregate stability, soil infiltration and water retention within AFS (Basche and DeLonge 2019; Cherubin et al. 2019; Ilstedt et al. 2007; Udawatta et al. 2008). Additionally, mineralisation of this additional soil organic matter, coupled with the deeper and more extensive root systems, may sustain efficient nutrient cycling and soil chemical processes (Cardinael et al. 2019; Sileshi et al. 2020). Nutrient availability can be further enhanced by rain throughfall from the tree canopy (Cana Verde et al. 2023; Perez‐Marin and Menezes 2008) or by the presence of nitrogen‐fixing trees (Sileshi 2016). The tree root safety net also was shown to reduce nutrient leaching and lixiviation, thereby improving water quality (Bergeron et al. 2011; Mayer et al. 2007; Zhu et al. 2020). Within AFS, trees can act as a physical barrier to wind and water flows, mitigating surface runoff and soil particle erosion (Du et al. 2022; Jia et al. 2019).

Collectively, these findings underscore the potential of AFS to secure and enhance soil health attributes, possibly to a greater extent than other agricultural interventions (Beillouin et al. 2023). Nevertheless, these positive outcomes are contingent on site and management conditions, and AFS may also involve important soil‐related trade‐offs and disservices. In particular, competition between trees and crops for water and nutrients can affect key soil properties, including soil moisture and nutrient availability, with strong spatial and temporal heterogeneity (Abdulai et al. 2018; Bayala and Prieto 2020; Cardinael et al. 2015; Kuyah et al. 2019), potentially resulting in reduced crop productivity (Abdulai et al. 2018; Ivezić et al. 2021). The balance between positive and negative effects of AFS depends primarily on tree species selection (Gosme et al. 2025; Rigal et al. 2022), management practices (Baier et al. 2023; Prasad et al. 2023; Wajja‐Musukwe et al. 2008), and pedoclimatic conditions (Hübner et al. 2021; Ngaba et al. 2024; Shi et al. 2018). The impacts of AFS also vary based on the land use in which they are implemented, i.e., cropland, grassland, or mixed livestock systems (Feliciano et al. 2018; Ma et al. 2020; Nair et al. 2021). Together, these elements point to the relevance of systematically synthesizing available evidence to identify the pedoclimatic conditions and AFS types under which soil‐related benefits are most prominent.

Field trials have provided detailed insights into specific or local soil processes in AFS, but their limited spatial scope hinders generalization of findings, whereas traditional literature reviews often lack robust quantitative synthesis and overlook knowledge gaps. In recent years, the number of field trials and meta‐analyses focusing on AFS has grown exponentially, encompassing studies of limited scope and varying quality (Beillouin et al. 2019; Köthke et al. 2022), underscoring the need to regularly compile and assess the evidence to provide up‐to‐date and robust quantification of AFS's effects on soil health attributes. Moreover, most meta‐analyses focus on one or a few soil parameters at a time. Yet, assessing soil health is inherently complex and requires the simultaneous consideration of multiple parameters (Lehmann et al. 2020), and the definition of soil health is dependent on the context and on the specific soil functions under consideration (Fierer et al. 2021). Consequently, evaluating the influence of AFS on a particular soil function necessitates the use of tailored soil metrics, rather than relying on a single, aggregated effect‐size to represent overall soil health. To address this complexity, we conducted a second‐order meta‐analysis synthesizing meta‐analyses to estimate precisely the overall quantitative AFS effect for multiple soil health attributes, while explicitly accounting for meta‐analysis' quality, redundancy of primary studies, and potential publication bias. Our objectives are therefore: (i) to present an overview of the quantitative effects of AFS on different aspects of soil health, based on the current state of knowledge and published literature; and (ii) to identify knowledge gaps in the effects of AFS on specific soil attributes.

We applied the iMAP rigorous methodological framework for conducting second‐order meta‐analyses (Makowski et al. 2021; Schievano et al. 2025), which enables a transparent and reproducible assessment of the effects of farming practices. Leveraging this framework allowed us to incorporate a broader range of meta‐analyses than previous global syntheses, resulting in a dataset that is both more comprehensive and up to date. Importantly, this study advances beyond the conventional pooling of data from existing meta‐analyses by explicitly addressing key sources of uncertainty, including primary‐study overlap, quality‐based weighting of effect‐sizes, and the use of bias‐ and heterogeneity‐adjusted meta‐analytical estimates. Second‐order meta‐analyses typically assume independence of effect‐sizes, yet overlapping primary studies can violate this assumption, increasing the risk of false‐positive results (Makowski et al. 2023). We systematically quantified and accounted for such redundancy, a step rarely implemented in prior syntheses. Another major source of imprecision in meta‐analyses is the presence of publication bias, arising from the preferential publication of statistically significant results or findings that align with study hypotheses. This bias can lead to inflated overall effect‐sizes and misrepresent the true impact of interventions (Bartoš et al. 2023). For the first time in the context of AFS, we not only estimate the presence of such bias but also mitigate its influence using robust Bayesian meta‐analysis (RobMA), integrating sensitivity analyses that assess heterogeneity and potential publication bias. By quantifying and integrating these sources of uncertainty, our approach strengthens the reliability of estimated effects and supports a critical appraisal of the existing body of evidence. Hence, we set out to comprehensively assess the impact of AFS on soil health attributes by addressing the following key questions:

1. What is the extent and robustness of the effects of AFS on soil health attributes compared to conventional, treeless agricultural systems, and how generalizable are these effects on a global scale?
2. How do different climate regions and AFS types influence the effects of AFS on soil health attributes?
3. Which effects of AFS on soil health attributes are robustly supported by current evidence, and where do critical knowledge gaps remain when accounting for systematic quality and bias adjustment?

## 2 Material and Methods

### 2.1 Meta‐Analysis Identification and Selection

The selection of meta‐analyses followed the PRISMA guidelines (Page et al. 2021). Potentially relevant studies were identified through systematic search in Scopus and Web of Science using the following query:Topic: (agroforestry OR agro‐forestry OR *silvo‐past* OR *silvopast* OR silvoarable OR silvo‐arable OR forest‐farm* OR food‐forest* OR agro‐silvicult* OR agrosilvicult* OR (alley AND crop*) OR agri‐silvicult* OR agrisilvicult*)ANDTopic:(metaanaly* OR meta‐analy* OR “systematic* review*” OR “evidence map” OR “global synthesis” OR “evidence synthesis” OR “research synthesis”) 


Searches were conducted in 2020, 2023 and June 2024. To ensure completeness, we also included meta‐analyses identified in a set of selected previous systematic reviews on agroforestry (Beillouin et al. 2019; Castle et al. 2022; Köthke et al. 2022). The study selection process followed the protocol set up by the JRC iMAP Farming practices Evidence Library (Schievano et al. 2024).

**FIGURE 1:**
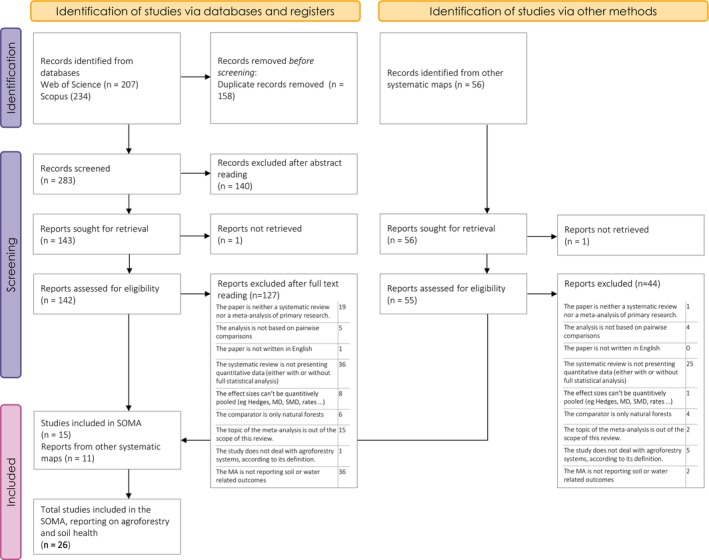
PRISMA flow diagram for the reporting of the identification and selection process of meta‐analyses included in our second‐order meta‐analysis (SOMA).

After removing duplicates, titles and abstracts were screened independently by four researchers. Full text was then evaluated against explicit exclusion criteria, as described in Schievano et al. (2024) (see also Figure 1). In addition, for inclusion, meta‐analyses had to (i) report soil‐related attributes and (ii) express effect‐sizes as ratios comparing each agroforestry intervention to its corresponding treeless agricultural controls. Any transformable ratio format (log‐transformed, percentage change, etc.) was considered. Following these criteria, 26 meta‐analyses were retained for synthesis (Table S1).

### 2.2 Data Extraction

The data were extracted using a standardized spreadsheet from the iMAP dataset (Schievano 2025; Schievano et al. 2024) by a single researcher to ensure consistency, and double‐checked by a second researcher to limit potential systematic errors. Quantitative effect‐sizes for each attribute were extracted from the meta‐analyses, and qualitative effects were subsequently inferred as positive, negative, no‐effect (not different from zero), or uncertain (not statistically tested). For quantitative effects, mean of the ratios and their corresponding measure of variability (standard errors, standard deviations, confidence intervals, or interquartile ranges) were extracted from the text, tables or from figures using ImageJ (Schneider et al. 2012). One meta‐analysis (Carey et al. 2020) did not provide measures of variability for all effect‐sizes. To address this, we imputed a confidence interval for the mean of ratio, i.e., the effect‐size, using the average 95% confidence interval derived from the corresponding soil attribute categories.

Additional relevant information, so‐called moderators, was collected from each meta‐analysis, including details on agroforestry practices and associated climatic zones. Based on available information, we classified the type of AFS as: silvoarable (SA), silvopastoral (SP), tree‐landscape feature (TLF), perennial shaded systems (PER) or unspecified AFS when insufficient detail was provided. For each AFS type, effect‐sizes were extracted exclusively relative to corresponding systems without trees. Silvoarable systems were compared only with treeless croplands; silvopastoral systems exclusively with treeless grasslands; perennial shaded systems with their unshaded perennial counterparts, and tree landscape features with equivalent systems lacking hedgerows or windbreaks. Consequently, each AFS type was associated with a single, clearly defined comparator, namely its treeless counterpart, which is why comparators were not further categorized within AFS subtypes. Climates were classified as either: arid, mediterranean, temperate or tropical according to the meta‐analysis authors' descriptions. Finally, the soil attributes were grouped into the following categories: soil organic carbon, soil biological quality, soil chemical quality, soil physical quality, soil water regulation, nutrient leaching & runoff, soil erosion (Table S2). The attribute classification was reviewed and validated by a panel of soil experts. Multiple attributes from the same study were considered as independent if they measured distinct soil processes or were reported for different specific AFS types or climates. When attributes were assessed at multiple soil depths, the total depth was prioritized when available; otherwise, measurements from the uppermost depth were considered.

### 2.3 Meta‐Analysis Quality Assessment and Primary Studies Overlap

The quality of each meta‐analysis was evaluated by at least two independent researcher following a standardized protocol (Schievano et al. 2024), comprising 16 independent quality criteria. These criteria assessed the comprehensiveness of the systematic review process (e.g., reporting the database or search string), the rigor of the data extraction and statistical analysis (e.g., detailed description of data extraction procedures and statistical methods), the reporting of effect‐sizes and their variability (e.g., inclusion of individual effect‐sizes and corresponding 95% confidence intervals), and the implementation of sensitivity analyses (see Figure S1, Table S3 or Schievano et al. (2024) for details). Each meta‐analysis received a quality score ranging from 0 to 1, reflecting the proportion of criteria fulfilled (Figure S1). The quality assessment of the 26 meta‐analyses included in our study shows that the consistency of reporting and the quality of statistical analysis of the studies varies greatly. The statistical methods employed in the meta‐analyses often lack robustness. Only 11 meta‐analyses explicitly reported evaluating publication bias, and only 14 assessed dataset heterogeneity. Furthermore, only half of the 26 meta‐analyses weighed individual effect‐sizes in their analyses (Figure S1). Notably, none of the studies reported bias‐adjusted effect‐sizes. Finally, only 58% of the meta‐analyses (15 out of 26) provided at least partial datasets alongside their publications, either through data repositories or as Supporting Information.

**FIGURE 2:**
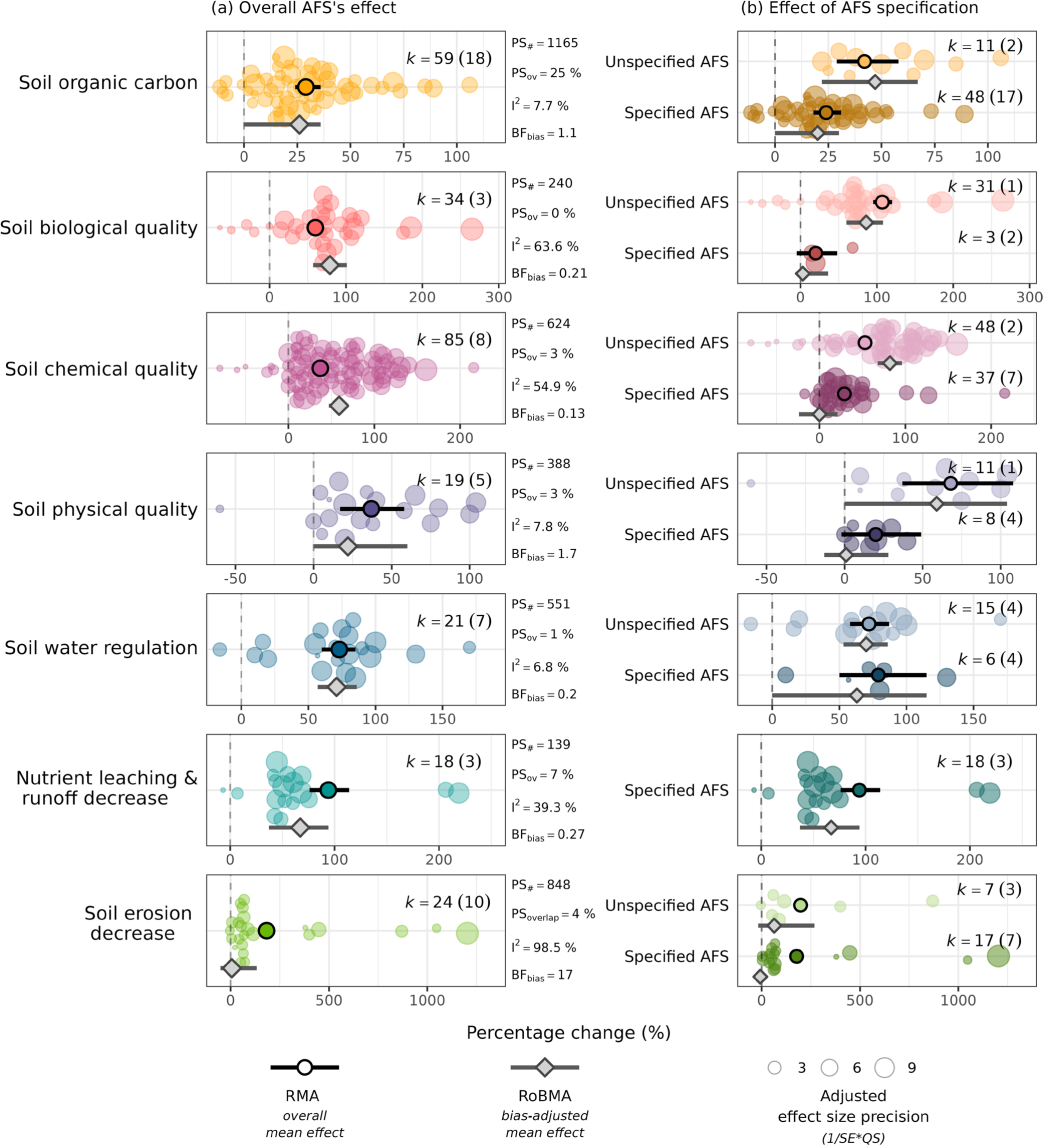
Mean effects of agroforestry compared with treeless agriculture on soil health attributes for the overall dataset (a) and depending on the presence of AFS specification (b). Opaque circles with black borders show overall mean effects calculated with random‐effect meta‐analysis (RMA). Grey diamonds represent the bias‐adjusted mean effects calculated with robust Bayesian meta‐analysis (RoBMA). Horizontal bars show the 95% confidence intervals (CI). Transparent dots show the individual effect‐size extracted from the meta‐analyses, with dot size reflecting adjusted precision—combining variance and the quality score of each meta‐analysis. *k* denotes the number of effect‐sizes included, while the number in parentheses indicates the number of meta‐analyses from which these effects were extracted. For the overall mean effect, additional metrics are reported: The number of primary studies (PS_#_), the proportion of overlapping primary studies (PS_ov_), the *I* ^2^ statistic estimating heterogeneity, and the BF_bias_ the evidence of publication bias.

We assessed the overlap of primary studies among the selected meta‐analyses. Of the 26 meta‐analyses considered, primary study lists were accessible for 22, leading to the exclusion of the remaining four from the overlap analysis (while all 26 meta‐analyses were retained for the quantitative synthesis). Missing bibliographic information's were retrieved using the Crossref metadatbase, accessed via an R pipeline implemented with the Rcrossref package (Chamberlain et al. 2025), with the detailed imputation process described in Schievano et al. (2025). Standardized bibliographic identifiers, preferably DOIs when available, were used to calculate the proportion of overlapping primary studies between each meta‐analysis pairs. Meta‐analysis subsets sharing more than 25% of their primary studies were identified using a pairwise comparison matrix (Figure S2). Following Lunny et al. (2021), all but one of the meta‐analyses exhibiting more than 25% overlap were excluded to evaluate whether overlap influenced overall mean effect estimates. The analysis was then repeated iteratively, each time retaining only one of the overlapping meta‐analyses. Across all parameters, total overlap among primary studies was 18%, most of which originated from soil organic carbon studies. A critical overlap (> 25%) was detected only for soil organic carbon, whereas no substantial overlap was identified for the six other soil attribute categories (Figure 2; Figure S2). Among the 15 meta‐analyses reporting on soil organic carbon with available primary study information, four showed more than 25% overlap between one another: Car18, Cha18, De18 and Ma20 (see Table S2 for meta‐analyses' reference). The overall estimate remained stable regardless of which meta‐analysis was included in the analysis, and publication bias was identified in all cases. Moderator analyses based on AFS type and climatic regions were similarly unaffected. Consequently, the effect‐sizes from all four overlapping meta‐analyses were retained in the analysis presented in this study. Four meta‐analyses did not provide primary study lists (Figure S1), limiting full verification of potential overlaps. Nevertheless, given that estimated overlap from available data is well below the critical threshold, it is unlikely that incorporating missing information would exceed this limit.

### 2.4 Meta‐Analytical Models and Sensitivity Analysis

For each attribute category, the overall mean effect‐size was estimated from independent effect‐sizes (*K*_*i*_) reported in the first‐order meta‐analyses. Effect‐sizes expressed as ratios were log‐transformed to stabilize variance and allow additive interpretation. Reported measures of uncertainty (standard errors, standard deviations, confidence intervals, or interquartile ranges) were harmonized and converted to standard deviations. Sampling variances were then calculated as:

$$v_{i}={{\mathrm{sd}}_{i}}^{2}/n_{i}\cdot {K_{i}}^{2}$$

where sd_*i*_ denotes the standardized uncertainty of *K*_*i*_, and *n*_*i*_ is the corresponding sample size. Each effect‐size was weighted by the inverse of its variance. The quality score of each corresponding meta‐analysis was used to adjust the weighing of individual *K* effect‐size, thereby reducing the influence of results derived from lower quality meta‐analyses (Doi et al. 2015). We first fitted random‐effects meta‐analytic models to account for unexplained heterogeneity among effect‐sizes. To model statistical dependence of multiple effect‐sizes derived from the same meta‐analysis, we specified a multilevel structure:

$$y_{\mathrm{ij}}=\mu +\mu_{j}+\varepsilon_{\mathrm{ij}},\mu_{j}∼N\;\left(0,\tau^{2}\right),\varepsilon_{\mathrm{ij}}∼N\;\left(0,v_{\mathrm{ij}}\right)$$

where *y*_*ij*_ is the log‐transformed effect‐size *i* from meta‐analysis *j*, *μ* the overall mean effect, *μ*_*j*_ the random deviation associated with meta‐analysis *j*, and *ε*_*ij*_ the sampling error with known variance *v*_*ij*_.

To assess whether effects varied systematically across contexts, moderator analyses were conducted by extending the model to include fixed effects for AFS type or climate:

$$y_{\mathrm{ij}}=\mu +\beta \cdot {\text{moderator}}_{\mathrm{ijj}}+\mu_{j}+\varepsilon_{\mathrm{ij}}$$

where the moderator_*ij*_ factor was defined as either the presence of an AFS classification, the specific type of AFS or the climate zone.

Uncertainty around pooled estimates was quantified with non‐parametric bootstrap confidence intervals (CI; 1000 resamples), avoiding distributional assumptions. Between‐study heterogeneity was evaluated with the Heterogeneity Q‐test and variance component *τ* ^2^ (Viechtbauer 2010). Publication bias was assessed using Egger's regression test, regression coefficient tests (Pustejovsky 2023), and visual inspection of funnel plot asymmetry.

As a complementary analytical framework, we implemented robust Bayesian meta‐analysis (RoBMA; Bartoš et al. 2023). This approach integrates multiple strategies to adjust effect‐size according to potential publication bias and heterogeneity of the data. RoBMA evaluates the presence or absence of an effect, heterogeneity, and publication bias using an ensemble of models. Each model's predictive performance is assessed using Bayes Factors (BF) that quantify the extent to which the observed data shift the odds from prior to posterior model probabilities. BF offers a continuous measure of evidence for or against the presence of an effect, heterogeneity, or publication bias. A BF greater than 1 suggests the presence of an effect/heterogeneity/bias, with BF values between 1 and 3 considered anecdotal evidence, 3–10 moderate evidence, and > 10 strong evidence. RoBMA outputs were used to assess the likelihood of heterogeneity and publication bias in the dataset, complementing classical sensitivity tests. Additionally, based on the estimated magnitude of the effect, heterogeneity, and publication bias, RoBMA provided a bias‐adjusted mean effect‐size, offering a more reliable estimation of the mean effect.

All statistical analyses were performed using the *metafor* and *RoBMA* packages in the R Software (Bartoš et al. 2023; R‐Core‐Team version 4.5 2021; Viechtbauer 2010). Figures were plotted using the *ggplot2* and *orchaRd* R packages (Nakagawa et al. 2023; Wickham 2016).

## 3 Results and Discussion

### 3.1 Extent and Robustness of Agroforestry Effects on Soil Health

#### 3.1.1 Soil Organic Carbon

Across meta‐analyses, AFS are consistently associated with higher soil organic carbon compared to treeless agriculture. Our random effect meta‐analysis (RMA) indicates a 29% _95% CI_[24%–36%] gain in soil organic carbon based on 59 effect‐sizes from 18 meta‐analyses compiling 1165 primary studies. This result aligns with previous second‐order syntheses on agroforestry (e.g., 20% _95% CI_[17%–23%] soil organic carbon increase in Beillouin et al. (2023) based on nine meta‐analyses), or in global re‐analyses of primary datasets (e.g., 32% _95% CI_[29%–35%] in Mathieu et al. (2025) based on 758 pairwise observations). In our dataset, when accounting for heterogeneity and publication bias through robust Bayesian meta‐analysis (RoBMA), the estimated mean effect is slightly smaller and statistically non‐significant (26% _95% CI_[0%–36%]), with confidence intervals overlapping zero (Figure 2). This occurs despite negligible evidence for heterogeneity and publication bias, as indicated by Bayes Factors (BF_hete_ = 0.57; BF_bias_ = 1.1), respectively.

Crucially, we show that the effect‐sizes are inflated when AFS type are not specified: meta‐analyses that aggregate all systems indiscriminately report larger soil organic carbon gains (47% _95% CI_[22%–67%]), i.e., more than double the mean effect of syntheses distinguishing specified AFS such as silvoarable, silvopastoral, or perennial practices (20% _95% CI_[0%–30%]; see Figure 2b). Restricting the dataset to specified systems reveals a modestly stronger presence of publication bias (BF_bias_ = 1.3 in specified vs. BF_bias_ = 0.55 in unspecified AFS), increasing uncertainty but not reversing positive trends. Based on the current evidence base, the most robust and scalable estimate of soil organic carbon gain in AFS corresponds to the bias‐adjusted effect in specified systems: 20% _95% CI_[0%–30%]; (Figure 2b).

#### 3.1.2 Soil Biological Quality

Evidence for agroforestry effects on soil biota remains too coarse to support generalization at functional or global scales. Among the three meta‐analyses (240 primary studies) reporting on biological attributes, RMA suggests a 60% _95% CI_[51%–69%] increase under AFS relative to treeless controls, whereas RoBMA, correcting for negligible publication bias (BF_bias_ = 0.21) and very strong heterogeneity (BF_hete_ = 1.1 × 10^14^), yields a slightly higher but more uncertain estimate (79% _95% CI_[57%–101%]). Within the unspecified AFS subset, soil biological quality is estimated to increase by 86% _95% CI_[60%–108%], while heterogeneity is markedly reduced (BF_hete_ = 6.2 × 10^9^) compared to the overall effect reported above. Given the current scarcity of data, the RoBMA bias‐adjusted effect of 86% increase likely represents the most reliable estimate of AFS impact on soil biology.

#### 3.1.3 Soil Chemical Quality

Soil chemical responses to agroforestry are, on the contrary, well characterized, with 85 effect‐sizes from 8 meta‐analyses basing their findings on 624 primary studies (Figure 2). RMA indicates a substantial mean increase of 37% _95% CI_[32%–43%], consistent with primary data syntheses reporting a 20% increase in soil fertility compared to treeless agricultural systems (Mathieu et al. 2025). Interestingly, our RoBMA analysis, accounting for pronounced heterogeneity (BF_hete_ = 3.2 × 10^17^) and small publication bias (BF_bias_ = 0.13), amplifies the estimated effect to 59% _95% CI_[47%–70%]. Critically, refining the analysis to specific AFS type substantially reduces the observed effect (Figure 2) and reveals a sharp increase in publication bias (BF_bias_ = 2.0 × 10^2^). The bias‐adjusted estimate for specified AFS becomes indistinguishable from zero (0% _95% CI_[−24%–21%]), whereas for unspecified AFS, the bias‐adjusted effect remains high (82% _95% CI_[68%–96%]) with markedly reduced heterogeneity (BF_hete_ = 4.0 × 10^2^) and publication bias (BF_bias_ = 0.16). The pronounced heterogeneity across these estimates underscores that the potential of AFS to enhance soil chemical quality is highly context‐dependent, and that deriving a robust global estimate will require deeper exploration of heterogeneity sources. Hence, the most robust estimate of AFS on soil chemical quality is hard to define at the moment but is most likely around the 59% RoBMA estimate given the range (−25% to 96%) of all our estimates.

#### 3.1.4 Soil Physical Quality

Soil physical parameters represent one of the least frequently synthesized dimensions of soil impact, with only 19 effect‐sizes from 5 meta‐analyses compiling 388 primary studies. AFS seem to have a positive overall effect of 37% _95% CI_[17%–58%] on soil physical properties (Figure 2). However, when heterogeneity (BF_hete_ = 0.68) and publication bias (BF_bias_ = 1.7) are accounted for, RoBMA reduces the mean estimate and renders it non‐significant (22% _95% CI_[0%–60%]). Disaggregating to specified AFS type further diminishes the effect and increases uncertainty (mean increase of 20% _95% CI_[2%–48%] in RMA and 1% _95% CI_[−13%–28%] in RoBMA, Figure 2b). This attenuation likely reflects the limited number of studies (8 effect‐sizes from 4 meta‐analyses) and a slight increase in publication bias within the subset (BF_bias_ = 3.3). Consequently, given the limited data availability, the overall RoBMA bias‐adjusted effect of 22% increase currently represents the most reliable estimate of AFS impacts on soil physical properties.

#### 3.1.5 Soil Water Regulation

Compared to other attributes, evidence for soil water regulation seems robust, even though it is based on only 21 effect‐sizes derived from seven meta‐analyses of 551 primary studies. Despite the relatively limited number of meta‐analyses, the effect appears stable regardless of the method implemented, as both RMA and RoBMA converge on nearly identical estimates, 73% _95% CI_[60%–85%] and 71% _95% CI_[57%–86%], respectively (Figure 2). Similar magnitudes have been reported by Mathieu et al. (2025), reinforcing the credibility of the observed effects. Our RoBMA analysis further confirms the strength of this evidence (BF_effect_ = 8.1 × 10^3^), as well as the negligible heterogeneity (BF_hete_ = 0.40) and publication bias (BF_bias_ = 0.20). Unlike soil organic carbon, chemical or physical properties, mean estimates for soil water regulation remain consistent regardless of whether AFS types are specified or not (Figure 2). Therefore, given this singular consistency across estimates and the still relatively limited dataset, the overall mean RoBMA bias‐adjusted effect of 71% currently represents the most robust and generalizable estimate of AFS impacts on soil water regulation.

#### 3.1.6 Nutrient Leaching and Runoff

Evidence on nutrient leaching and runoff is extremely limited, with only three meta‐analyses reporting 18 aggregated effect‐sizes based on 139 primary studies. All effects were reported specifying the AFS type. Despite this narrow evidence base, both RMA and RoBMA point to large leaching reductions under agroforestry: 94% _95% CI_[76%–114%] and 67% _95% CI_[37%–94%] decrease, respectively. Publication bias appears negligible (BF_bias_ = 0.27), and heterogeneity remains moderate (BF_hete_ = 12, see Figure 2). Thus, the RoBMA bias‐adjusted mean effect of 67% likely represents the most reliable estimate of AFS's capacity to mitigate nutrient leaching.

#### 3.1.7 Soil Erosion

Soil erosion control by AFS was addressed in 10 meta‐analyses, drawing on 848 primary studies. The conventional model estimates very large benefits, with RMA suggesting a 183% _95% CI_[171%–195%] improvement. However, this signal collapses once publication bias and heterogeneity are accounted for. Both are extreme (BF_bias_ = 17, BF_hete_ = ∞, Figure 2), and RoBMA reduces the mean effect to 6% _95% CI_[−52%–132%], indicating that the apparent benefit is highly uncertain. Crucially, specifying AFS type does not reduce heterogeneity or bias, implying that the variance is not attributable to management type alone. The extreme heterogeneity does not indicate weak agroforestry effects but probably reflects the context in which most erosion studies are conducted: high‐risk settings with steep slopes, degraded soils or intense rainfall, where large effects are both more detectable and more publishable. This sampling bias might inflate mean estimates and drives variance, a pattern consistent with the global concentration of erosion occurrence and measurement in vulnerable landscapes (Borrelli et al. 2017; Panagos et al. 2021; Vanmaercke et al. 2015) and its sensitivity to rainfall extremes and land degradation (García‐Ruiz et al. 2015; Poesen 2018). Without accounting for moderators such as rainfall erosivity, slope, soil type or vegetation structure, meta‐analyses conflate intervention effects with baseline vulnerability. Correcting for selective reporting and stratifying by pedoclimatic context is thus essential to derive scalable estimates across global contexts; a task we were unable to accomplish due to insufficient information in most of the synthesized meta‐analyses.

### 3.2 Influence of AFS Types and Climate Regions on Soil Health Attributes

**FIGURE 3:**
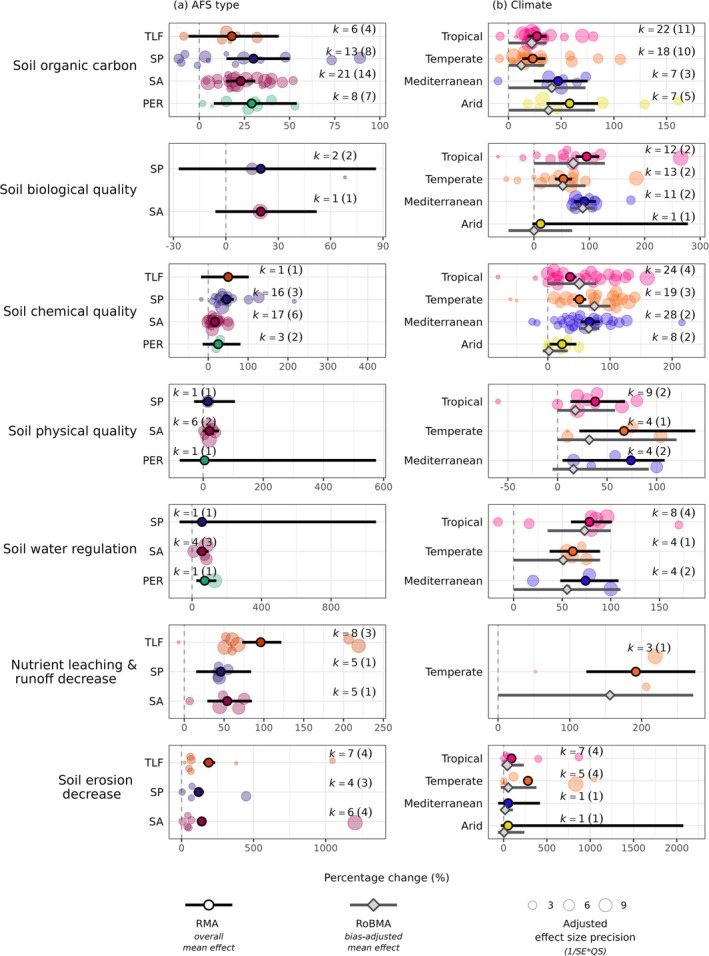
Mean effects of agroforestry compared with treeless agriculture for different AFS types (a) and climate regions (b). Opaque circles with black borders show overall mean effects calculated with random‐effect meta‐analysis (RMA). Grey diamonds represent the bias‐adjusted mean effects calculated with robust Bayesian meta‐analysis (RoBMA). Horizontal bars show the 95% confidence intervals (CI). Transparent dots show the individual effect‐size extracted from the meta‐analyses, with dot size reflecting adjusted precision—combining variance and the quality score of each meta‐analysis. *k* denotes the number of effect‐sizes included, while the number in parentheses indicates the number of meta‐analyses from which these effects were extracted. The specified AFS types are: Tree‐Landscape Features (TLF), Silvopastoral systems (SP), Silvoarable systems (SA), Perennial agroforestry (PER).

We demonstrated that for all investigated soil attributes, effect sizes do not differ across AFS types (Figure 3), but this likely reflects insufficient disaggregation rather than genuine consistency. Refining the analysis to specific AFS types thus fails to explain the sources of heterogeneity present in AFS effects on soil health attributes. Exploring continuous descriptors of AFS characteristics, such as AFS's age, tree density, or functional diversity, could help address this limitation while also mitigating the lack of standardized definitions of agroforestry practices in the literature.

Soil organic carbon in AFS tends to increase more strongly under conditions of greater climatic aridity. Specifically, soil organic carbon increased by 58% _95% CI_[36%–85%] in arid regions, followed by 47% _95% CI_[24%–75%] in mediterranean climates, 27% _95% CI_[18%–36%] in tropical, and 23% _95% CI_[13%–35%] in temperate regions based on RMA (Figure 3). These findings are consistent with previous research indicating that arid climates amplify the beneficial effects of AFS on soil organic carbon accumulation (Mathieu et al. 2025; Pan et al. 2025). Our RoBMA analyses corroborates this pattern, albeit with slightly lower bias‐adjusted estimates (Figure 3), and reveals minimal publication bias and heterogeneity. Again, the bias‐adjusted confidence intervals all intersect with zero, suggesting that conventional meta‐analyses may overestimate the extent to which AFS enhance soil organic carbon across different climatic zones.

In mediterranean climates, estimates of the positive effect of AFS for both biological and chemical soil properties appear robust and reliable. This is supported by consistently negligible heterogeneity and publication bias (BF_hete_ & BF_bias_ < 1), and by the close alignment between RoBMA bias‐adjusted and RMA estimates (Figure 3). In contrast, substantial heterogeneity was observed in tropical and temperate subgroups. A more comprehensive exploration of the sources of heterogeneity, using continuous variables such as precipitation, temperature, soil texture, spatial and temporal gradient, could be achieved if more meta‐analyses made their datasets publicly accessible.

### 3.3 Knowledge Gaps, Limitations and Outlook

**FIGURE 4:**
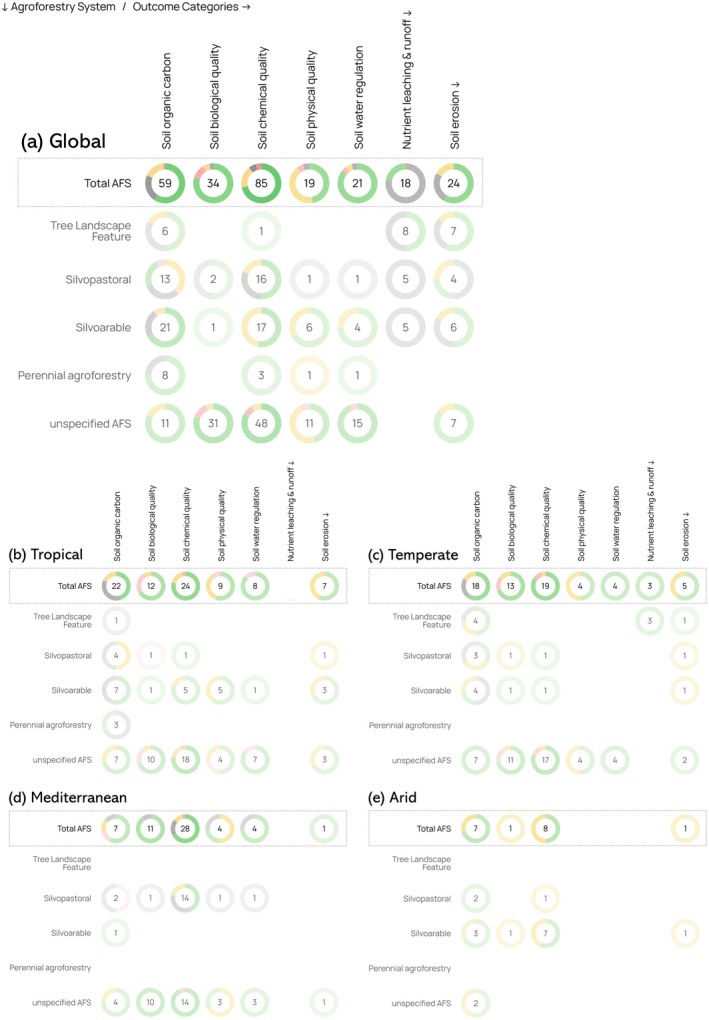
Qualitative effects of agroforestry systems (AFS) on soil and water related attributes retrieved from the 26 meta‐analyses globally (a), in Tropical (b), Temperate (c), Mediterranean (d), and Arid (e) climate regions. The numbers in the donut indicate the number of independent observations. The donut segment indicates the proportion of positive (green), negative, (red), uncertain (yellow) and non‐statistically tested (grey) results. The first line shows the total number of effects extracted for all AFS types, and the bottom five lines show the number of effects extracted for each AFS type. The symbol ↓ behind *“nutrient leaching & runoff”* and “*soil erosion*” stands for *decrease*.

Our synthesis delivers compelling evidence of agroforestry's potential to enhance soil health, yet substantial gaps persist in the evidence base, particularly regarding process‐explicit indicators and comprehensive coverage of soil attributes across biomes. A major research gap concerns the impact of AFS on soil biological properties, which is critical for understanding their role in nutrient and carbon cycling. Two of the three meta‐analyses reporting on soil biology combine multiple metrics and processes into aggregated indices (“soil biota” or “soil activity and diversity”; Car20 and Vis24, see Table S2), limiting ecological interpretability and precluding any inference about functional turnover, nutrient cycling, or carbon stabilization. This limitation is aggravated by the fact that only one meta‐analysis extends beyond regional datasets focusing on California and Latin America, and 90% of the extracted effect‐sizes originate from studies that do not identify the AFS type. Consequently, no global meta‐analytic evidence exists to resolve the responses of silvopastoral, silvoarable, or perennial systems on soil biology, despite mechanistic expectations of divergent belowground pathways via litter stoichiometry, rhizosphere interactions, and mycorrhizal networks (Battie‐Laclau et al. 2020; Cardinael et al. 2020; Guillot et al. 2019). Current syntheses therefore conflate signal and noise, providing effect‐size estimates disconnected from biological functions, spatial representativeness, and management context. Transitioning to trait‐based, process‐explicit indicators and biome‐explicit sampling is essential to meaningfully integrate soil biology into global assessments of agroforestry's role in carbon–nutrient–microbe couplings (Ding et al. 2022; Fierer et al. 2021). As with soil biology, global coverage is also limited for soil physical parameters. Aside from two global meta‐analyses (Kim16, Nga24), most data are regionally constrained to California (Car20), China (Sun18), or the humid tropics (Muc20), limiting the ability to infer AFS effects on physical properties across biomes. Another key gap concerns the differential contributions of AFS to water regulation across climatic zones and management types (Figures 3 and 4). These dimensions are critical for understanding how agroforestry enhances agroecosystem resilience under climate change, as they underpin mechanisms that buffer increasingly frequent and severe climatic disturbances. This study also underscores the lack of resolution regarding AFS effects on nutrient leaching across climatic regions. Current specifications exist only for temperate zones (Figure 4c), revealing a major geographic blind spot at a time when nutrient losses are projected to intensify in agricultural systems of the Global South. Data availability for arid climates remains limited across all soil and water attributes (Figure 4e), representing a significant gap, particularly given that afforestation is a key nature‐based solution proposed to combat desertification (FAO 2017; Sileshi et al. 2023). No data were explicitly reported for boreal or continental climates. Owing to the current uneven geographical coverage, global assessments of AFS should be interpreted with caution, as highlighted in evidence maps of agroforestry research (Beillouin et al. 2019; Köthke et al. 2022; Mathieu et al. 2025). This limitation underscores the need to address existing knowledge gaps in order to refine our understanding of AFS effects on soils within specific pedoclimatic contexts and to support the formulation of robust, generalizable conclusions on their global benefits.

The apparent absence of differential effects among AFS type or climatic zone likely reflects limitations in the primary and meta‐analytic evidence rather than true biological equivalence. Furthermore, explicit reporting on perennial shaded AFS remains scarce, despite their ubiquity in tropical regions and their status as one of the most widespread and well‐studied systems globally (Mathieu et al. 2025). This gap suggests that many of these effects may be concealed within non‐specified effect sizes. Most meta‐analyses aggregate heterogeneous practices, lack standardized definitions, and fail to clearly delineate interventions and comparators, thereby obscuring practice‐specific responses. For example, in the case of alley cropping, persistent ambiguity remains regarding whether the tree strip was considered the agroforestry intervention and compared to a treeless field, whether the cropped alley adjacent to trees was used as the intervention, or whether the control was located within the alley or in a separate treeless area. Moreover, there is widespread uncertainty about whether the different components of the AFS (e.g., the cropped alley and the tree strip) were averaged based on their relative area to estimate the overall agroforestry effect. Empirical evaluation of primary studies reinforces this concern: Minarsch et al. (2024) found that only one‐third of publications specified sampling positions within the AFS; just three out of 23 employed designs capable of capturing tree‐induced effects on soil organic carbon, and ten studies did not sample tree strips at all. These methodological gaps constrain the detection of nuanced effects, inflate uncertainty, and limit the reusability of the data from primary studies.

Mechanistically, existing evidence suggests that improvements in soil health attributes are primarily driven by the presence of trees or perennial components (Romero et al. 2024; Saviozzi et al. 2001). However, continuous factors such as system age, tree density, species diversity, or spatial gradients remain largely unexplored, with only 11 meta‐analyses considering these variables. Additionally, agroforestry is often implemented alongside other soil conservation practices, such as reduced mechanized tillage, cover cropping, organic amendments, and residue retention (Branca et al. 2013), although the extent of this co‐adoption by farmers has not yet been quantified. This raises important questions regarding interactions among practices and their combined effects on soil health, a dimension that is not yet adequately captured by meta‐analyses, which predominantly rely on primary studies comparing single, clearly defined interventions. Addressing these gaps will require the adoption of standardized definitions, consistent sampling protocols, and explicit consideration of structural and compositional continuous covariates, as highlighted by Minarsch et al. (2025) and Cardinael et al. (2025), to enable robust, practice‐specific insights.

### 3.4 Risk of Overestimation of AFS Effect Due to Publication Bias and Aggregation

Our findings highlight that uncorrected second‐order syntheses can inflate effect sizes through overlap and selective reporting, echoing concerns raised by Makowski et al. (2023). Importantly, this study is the first to explicitly quantify inflation resulting from bias‐unadjusted approaches, revealing that traditional meta‐analytic estimates of AFS's contribution to certain soil parameters may be overstated (Figure 2). For example, soil organic carbon estimates may be inflated by 1%–19% depending on the case; a scale sufficient to alter sequestration assessments at policy‐relevant thresholds (Lugato et al. 2014). In addition, when bias‐adjustment methods are applied, the confidence interval for AFS's effect on soil organic carbon expands and overlaps zero, indicating that improvements in soil organic carbon might be far more variable than traditionally reported and likely highly context‐dependent. The need for such large‐scale corrections is underscored in other scientific domains by broad empirical evidence showing that published meta‐analyses frequently suffer from inflated effect sizes. For instance, Sterne et al. (2000) demonstrated that excluding unpublished data can lead to an average 15% overestimation in clinical effect sizes, while the meta‐meta‐analysis by Bartoš et al. (2024) established that a significant proportion of meta‐analyses across all disciplines exhibit clear signs of selection bias. Furthermore, even high‐quality resources, such as the Cochrane Database, show clear evidence of bias in a significant number of syntheses (Kicinski et al. 2015).

The risk of effect overestimation may also arise from aggregating heterogeneous interventions. In our analysis, combining unspecified systems without accounting for management type consistently inflated AFS impact estimates. The discrepancy between specified and unspecified AFS estimates is unlikely to reflect true biological differences; rather, it stems from methodological and reporting limitations. Pooling meta‐analyses that do not specify AFS types can inflate estimated effects, as shown by Geissbühler et al. (2021) and Kaufmann et al. (2016), who demonstrated that aggregating heterogeneous interventions biases effect‐sizes. Li et al. (2025) further showed that such aggregation increases uncertainty and can misrepresent intervention outcomes, highlighting the need for system‐specific analyses.

These findings underscore that even where meta‐analytical coverage is extensive—as for soil organic carbon or soil chemical quality—conventional estimates can be misleading; rigorous bias corrected, and system‐specific syntheses are required to constrain agroforestry's true potential to enhance soil health and guide evidence‐based management and policy.

## 4 Conclusion

In this study, we provide, for the first time, robust quantitative estimates of agroforestry's effects on key soil properties, underscoring the considerable potential of agroforestry systems (AFS) to improve soil health. Overall, when accounting for meta‐analytic quality and potential bias, AFS demonstrate statistically significant improvements in soil chemical quality (59%) and water regulation (71%), as well as in biological quality (86%), although the latter is characterized by substantial heterogeneity. By contrast, effects on other soil attributes are less explicit: the estimated 20% increase in soil organic carbon, together with mean improvements in soil physical quality (22%) and erosion control (6%), remain statistically non‐significant and therefore warrant cautious interpretation. Moreover, the current evidence base may overstate certain effects, particularly those related to soil organic carbon, chemical properties, and erosion decrease, due to publication bias, selective reporting, and the aggregation of heterogeneous management practices. As global syntheses rarely incorporate bias adjustment procedures, such overestimations may extend beyond AFS to other agricultural practices as well. Consequently, inflation of carbon sequestration estimates risks distorting the perceived potential of agroecosystems to mitigate climate change.

Although bias‐adjusted confidence intervals frequently overlap with zero, our findings indicate that AFS tend to exert positive effects on soils across all climatic zones, underscoring their multifunctionality in diverse pedoclimatic contexts. Notably, agroforestry shows particularly strong potential under arid and mediterranean conditions, reaffirming its critical role in combating landscape desertification and enhancing the resilience of agroecosystems globally. Nevertheless, the uneven geographical coverage of existing AFS evidence calls for caution in current global assessments and reinforces the need for further, geographically balanced investigations.

Agroforestry encompasses a wide array of agricultural practices implemented across highly diverse socio‐environmental contexts. Thus meaningful, equitable global assessments of its impacts require careful consideration of this diversity and the varied motivations behind their adoption and maintenance. Currently, more than half of the existing syntheses on AFS effects on soil lack transparency in data reporting, underscoring the need for future research to adopt standardized sampling and reporting protocols capable of capturing AFS's diversity. Enhancing data reusability will facilitate a more comprehensive exploration of the drivers behind AFS effects and enable a fuller understanding of their benefits for soil health across diverse climatic, pedological, and sociocultural settings. Updating our knowledge of AFS potential, especially in areas where knowledge remains limited, is essential for fostering adaptive and socially relevant research that meets contemporary challenges.

## Author Contributions

**Rubeaud Camille Manon:** conceptualization, formal analysis, investigation, writing – original draft. **Six Johan:** supervision, writing – review and editing. **Kay Sonja:** funding acquisition, investigation, writing – review and editing. **Walder Florian:** writing – review and editing. **Köthke Margret:** investigation, writing – review and editing. **Beillouin Damien:** formal analysis, writing – review and editing. **Schievano Andrea:** investigation, writing – review and editing, data curation.

## Funding

This work was supported by the DigitAF project (grant agreement no. 101059794), co‐funded by the European Commission, European Research Agency, within the Horizon Europe program. The views and opinions expressed in this report are purely those of the writers and may not in any circumstances be regarded as stating an official position of the European Commission.

## Conflicts of Interest

The authors declare no conflicts of interest.

## Supporting information


**Table S1:** List of meta‐analyses (MA) included in our study. The table shows the soil outcome category extracted in each MA and indicates whether information about AFS type and climate zone was provided.
**Table S2:** Classification of the soil parameters into categories.
**Table S3:** Quality criteria used for the assessment of meta‐analyses' quality.
**Table S4:** Summary of the statistical and sensitivity analysis.
**Figure S1:** Quality assessment of each meta‐analysis and final quality score used for adjusting the weighing of the meta‐analysis' effect‐size.
**Figure S2:** Pairwise comparison matrix of the proportion of primary studies (PS) shared between meta‐analysis (MA) for the 22 meta analyses reporting their PS.

## Data Availability

The data used in this article are published here: https://doi.org/10.5281/zenodo.20036728.
