# Supplementary material for: Global Bias‐Aware Synthesis of Meta‐Analyses Reveals Agroforestry's Potential for Improving Soil Health
Source: Glob Chang Biol. 2026 Jun 18;32(6):e70960. doi: 10.1111/gcb.70960 (PMC13280171; doi:10.1111/gcb.70960)
Supplement: Supplementary file 1 — Table S1: List of meta‐analyses (MA) included in our study. The table shows the soil outcome category extracted in each MA and indicates whether information about AFS type and climate zone was provided. Table S2: Classification of the soil parameters into categories. Table S3: Quality criteria used for the assessment of meta‐analyses' quality. Table S4: Summary of the statistical and sensitivity analysis. Figure S1: Quality assessment of each meta‐analysis and final quality score used for adjusting the weighing of the meta‐analysis' effect‐size. Figure S2: Pairwise comparison matrix of the proportion of primary studies (PS) shared between meta‐analysis (MA) for the 22 meta analyses reporting their PS. [file GCB-32-e70960-s001.pdf]

Supplementary Information

Table S1: List of meta-analyses (MA) included in our study. The table shows the soil outcome category extracted in each MA and indicates whether information about AFS type and climate zone was provided.

						Soil outcome categories							*Info about:*	
	ID	Autohors	Year	Title	Doi	Soil organic Carbon	Soil biological quality	Soil chemical quality	Soil physical quality	Soil water regulation	Nutrient leaching	Erosion	AFS type	Climate
1.	Bas19	Basche & DeLonge	2019	Comparing infiltration rates in soils managed with conventional and alternative farming methods: A meta-analysis	https://doi.org/10.1371/journal.pone.0215702					×				
2.	Car18	Cardinael et al.	2018	Revisiting IPCC Tier 1 coefficients for soil organic and biomass carbon storage in agroforestry systems	https://doi.org/10.1088/1748-9326/aaeb5f	×							×	×
3.	Car20	Carey et al.	2020	Supporting evidence varies for rangeland management practices that seek to improve soil properties and forage production in California	https://doi.org/10.3733/ca.2020a0015	×	×	×	×	×			×	×
4.	Cha18	Chatterjee et al.	2018	Changes in soil carbon stocks across the Forest-Agroforest-Agriculture/Pasture continuum in various agroecological regions: A meta-analysis	https://doi.org/10.1016/j.agee.2018.07.014	×							×	×
5.	De18	De Stefano & Jacobson	2018	Soil carbon sequestration in agroforestry systems: a meta-analysis	https://doi.org/10.1007/s10457-017-0147-9	×							×	
6.	Dre21	Drexler et al.	2021	Carbon sequestration in hedgerow biomass and soil in the temperate climate zone	https://doi.org/10.1007/s10113-021-01798-8	×							×	×
7.	Du22	Du et al.	2022	Conservation management decreases surface runoff and soil erosion	https://doi.org/10.1016/j.iswcr.2021.08.001							×		
8.	Ils07	Ilstedt et al.	2007	The effect of afforestation on water infiltration in the tropics: A systematic review and meta-analysis	https://doi.org/10.1016/j.foreco.2007.06.014					×				×

9.	Ive22	Ivezić et al.	2022	Soil Organic Carbon in Alley Cropping Systems: A Meta-Analysis	https://www.mdpi.com/2071-1050/14/3/1296	×							×	×
10.	Jia19	Jia et al.	2019	Effects of minimum soil disturbance practices on controlling water erosion in China's slope farmland: A meta-analysis	https://doi.org/10.1002/ldr.3258							×		
11.	Kim16	Kim et al.	2016	Carbon sequestration and net emissions of CH4 and N2O under agroforestry: Synthesizing available data and suggestions for future studies	https://doi.org/10.1016/j.agee.2016.04.011	×		×	×				×	
12.	Kum23	Kumara et al.	2023	Carbon sequestration potential of agroforestry systems in Indian agricultural landscape: A Meta-Analysis	https://doi.org/10.1016/j.ecoser.2023.101537	×							×	×
13.	Kuy19	Kuyah et al.	2019	Agroforestry delivers a win-win solution for ecosystem services in sub-Saharan Africa. A meta-analysis	https://doi.org/10.1007/s13593-019-0589-8	×		×		×		×	×	×
14.	Ma20	Ma et al.	2020	Carbon accumulation in agroforestry systems is affected by tree species diversity, age and regional climate: A global meta-analysis	https://doi.org/10.1111/geb.13145	×							×	×
15.	May07	Mayer et al.	2007	Meta-Analysis of Nitrogen Removal in Riparian Buffers	https://doi.org/10.2134/jeq2006.0462						×		×	×
16.	Muc20	Muchane et al.	2020	Agroforestry boosts soil health in the humid and sub-humid tropics: A meta-analysis	https://doi.org/10.1016/j.agee.2020.106899	×		×	×	×		×	×	×
17.	Nga24	Ngaba et al.	2024	Meta-analysis unveils differential effects of agroforestry on soil properties in different zonobiomes	https://doi.org/10.1007/s11104-023-06385-w	×	×	×	×	×		×		×
18.	Shi18	Shi et al.	2018	Agroforestry systems: Meta-analysis of soil carbon stocks, sequestration processes, and future potentials	https://doi.org/10.1002/ldr.3136	×							×	×
19.	Sil16	Sileshi	2016	The magnitude and spatial extent of influence of Faidherbia albida trees on soil properties and primary productivity in drylands	https://doi.org/10.1016/j.jaridenv.2016.03.002	×		×					×	×

20.	Sun18	Sun et al.	2018	The effects of land use change on soil infiltration capacity in China: A meta-analysis	https://doi.org/10.1016/j.scitotenv.2018.01.104	×			×	×			×	
21.	Tor16	Torralba et al.	2016	Do European agroforestry systems enhance biodiversity and ecosystem services? A meta-analysis	https://doi.org/10.1016/j.agee.2016.06.002			×				×	×	×
22.	Tya24	Tyagi & Haritash	2025	Climate-smart agriculture, enhanced agroproduction, and carbon sequestration potential of agroecosystems in India: a meta-analysis	https://doi.org/10.1007/s13412-024-00917-1	×							×	×
23.	Van17	Van Vooren et al.	2017	Ecosystem service delivery of agri-environment measures: A synthesis for hedgerows and grass strips on arable land	https://doi.org/10.1016/j.agee.2017.04.015	×					×	×	×	×
24.	Vis24	Visscher et al.	2024	Agroforestry enhances biological activity, diversity and soil-based ecosystem functions in mountain agroecosystems of Latin America: A meta-analysis	https://doi.org/10.1111/gcb.17036	×	×	×				×	×	×
25.	Xio18	Xiong et al.	2018	Effects of soil conservation techniques on water erosion control: A global analysis	https://doi.org/10.1016/j.scitotenv.2018.07.124							×	×	
26.	Zhu20	Zhu et al.	2020	Reductions in water, soil and nutrient losses and pesticide pollution in agroforestry practices: a review of evidence and processes	https://doi.org/10.1007/s11104-019-04377-3						×	×	×	

Table S2: Classification of the soil parameters into categories

Category	Soil outcome	Meta-analysis' ID in which the outcome was retrieved
Soil Carbon	Soil Organic Carbon (SOC) content	Kuy19, Ive22, Muc20, Nga24, Sil16
	SOC stock	Car18, Car20, Cha18, De 18, Dre21, Kum23, Ma 20, Nga24, Shi18, Tya24, Van17
	SOC concentration	Kim16, Nga24
	Soil Organic Matter (SOM) content	Nga24, Sun18
	Belowground Biomass C stock	Vis24
Soil Biological Quality	Bacterial community abundance	Nga24
	Beta- Glucosidase activity	Nga24
	Fungal community abundance	Nga24
	Microbial biomass carbon	Nga24
	Microbial biomass nitrogen	Nga24
	Microbial biomass phosphorus	Nga24
	Microbial community abundance	Nga24
	Soil activity and diversity	Vis24
	Soil biota	Car20
	Soil Basal respiration	Nga24
	Soil respiration	Nga24
	Protease activity	Nga24
	Urease activity	Nga24
Soil Chemical Quality	Base saturation	Car20
	Cation	Car20
	Dissolved organic N	Nga24
	N cycling potential	Car20
	Soil available Ca	Car20, Nga24, Sil16
	Soil available K	Car20, Nga24, Sil16
	Soil available Mg	Nga24, Sil16
	Soil available Mn	Car20, Nga24
	Soil available P	Kuy19, Muc20, Nga24, Sil16
	Soil C/N decrease	Car20
	Soil CEC	Car20, Nga24
	Soil electrical conductivity	Nga24
	Soil Fe2+	Nga24
	Soil fertility/nutrient cycling parameters	Tor16
	Soil mineral N	Car20, Muc20, Nga24
	Soil Na	Car20
	Soil nutrient provisioning	Vis24
	Soil Olsen P	Nga24
	Soil Organic N	Nga24
	Soil P	Car20
	Soil pH	Car20, Muc20, Nga24, Sil16
	Soil total N	Car20, Kim16, Kuy19, Muc20, Nga24, Sil16
	Soil total P	Nga24
	Soil Zn2+	Nga24

Soil Physical Quality	Soil aggregates mean Weight Diameter	Muc20, Nga24
	Soil bulk density decrease	Kim16, Nga24, Sun18
	Soil C-aggregate	Muc20
	Soil decompaction	Car20
	Soil macroaggreagate	Muc20
	Soil N-aggreagate	Muc20
	Soil porosity	Muc20, Nga24
	Water stable aggregate	Nga24
Soil Water Regulation	Infiltration capacity	Ils07
	Infiltration rate	Kuy19
	Initial infiltration rate	Bas19, Sun18
	Soil moisture	Kuy19, Nga24
	Soil Water Content	Car20, Nga24
	Steady-state infiltration rate	Sun18
	Water holding capacity	Nga24
	Water infiltration	Muc20, Nga24
	Water regulation (infiltration and moisture)	Kuy19
Nutrient leaching and runoff - Water Quality	Ca, Mg, etc loss reduction caused by leaching, runoff and soil losses	Zhu20
	K loss reduction caused by leaching, runoff and soil losses	Zhu20
	N interception: surface and subsurface	Van17
	N loss reduction caused by leaching, runoff and soil losses	Zhu20
	Organic carbon stock loss reduction with leaching and water erosion	Zhu20
	P interception: surface	Van17
	P loss reduction caused by leaching, runoff and soil losses	Zhu20
	Water Nitrate concentration	May07
Soil Erosion	Sediment production reduction	Jia19
	Soil erosion control	Du22, Muc20, Nga24, Tor16, Vis24, Xio18, Zhu20
	Soil loss reduction	Kuy19
	Soil runoff control	Du22, Jia19, Kuy19, Muc20, Van17, Xio18, Zhu20

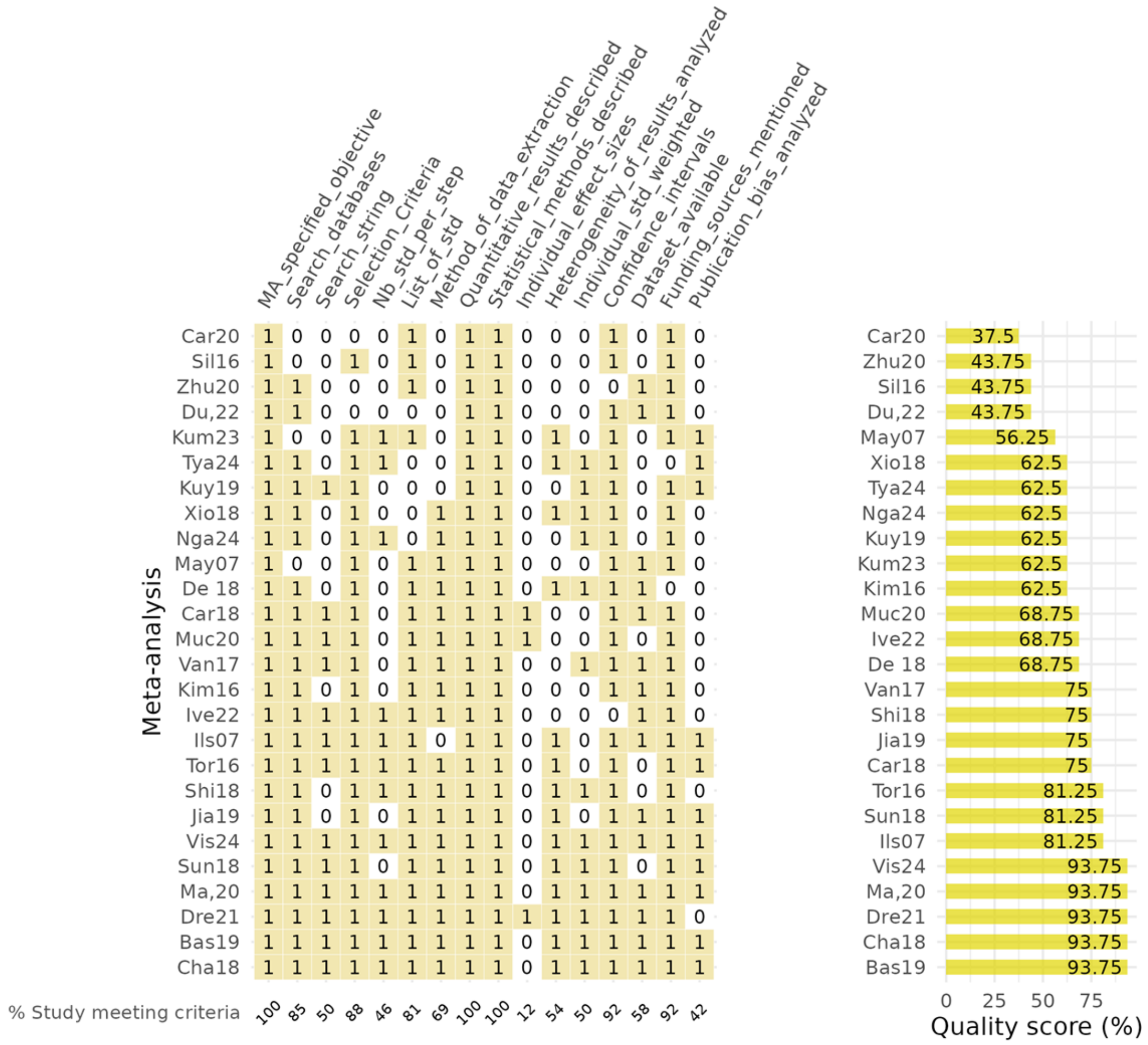

Figure S1: Quality assessment of each meta-analysis and final quality score used for adjusting the weighing of the meta-analysis' effect-size

Table S3: Quality criteria used for the assessment of meta-analyses' quality

Scoping	1	The objectives of the synthesis are clearly stated
Search	2	The search databases are clearly mentioned
	3	The search string(s) is/are clearly reported in details
Study selection	4	The selection criteria are mentioned and clearly explained
	5	All details of the selection process are described (e.g. Prisma statement)
	6	The list of selected studies is reported and complete of full references.
Data extraction	7	The methods used for data selection, extraction and storage is clearly explained.
Statistical analysis	8	A quantitative assessment of the effects is presented, complete of proper statistics.
	9	The statistical methods are clearly described.
	10	Individual effect sizes of primary-studies comparisons are reported (e.g. forest plot or tables).
	11	Heterogeneity of the effects is analyzed.
	12	Individual studies or experiments are weighted.
	13	Confidence intervals are presented.
Transparency and bias	14	The primary-studies dataset is made available and accessible.
	15	The funding sources are reported.
	16	The publication bias was analyzed.

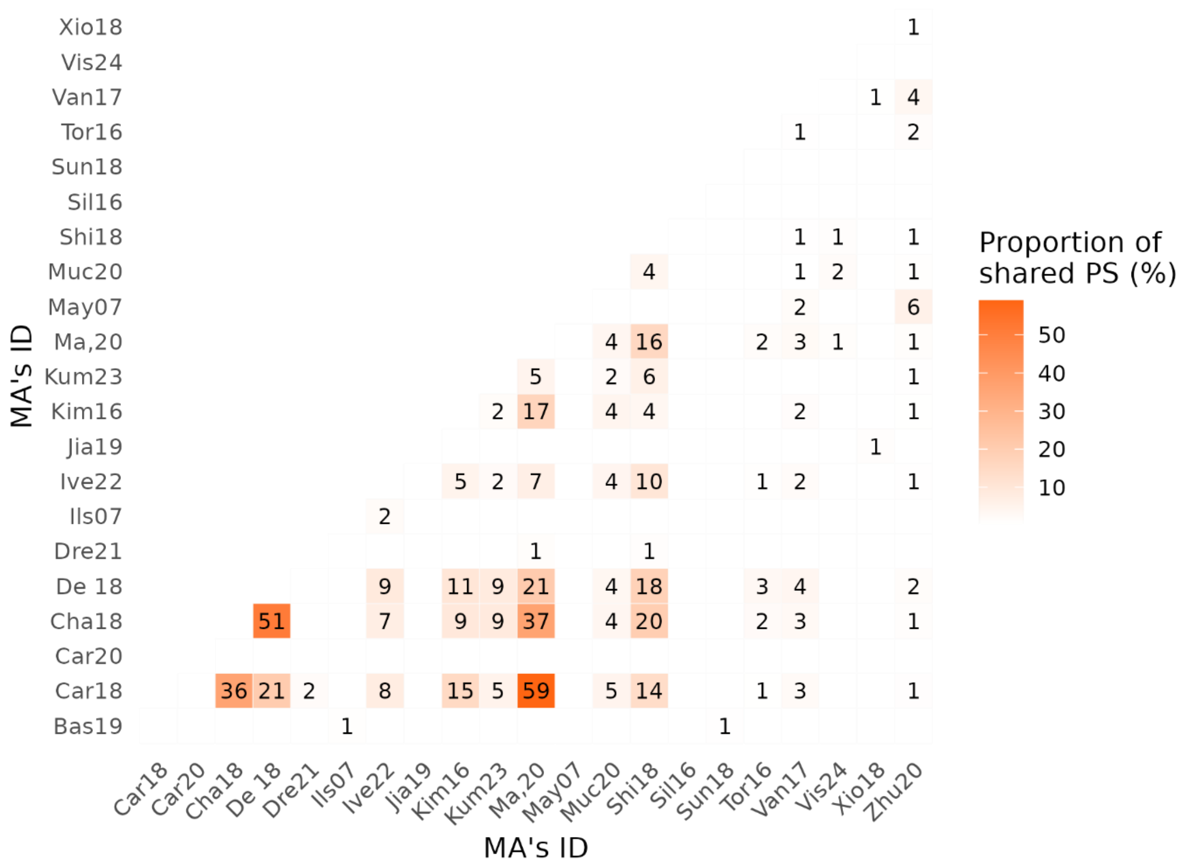

Figure S2: Pairwise comparison matrix of the proportion of primary studies (PS) shared between meta-analysis (MA) for the 22 meta-analyses reporting their PS

Table S4: summary of the statistical and sensitivity analysis

	RoBMA									
	BF values			bias-adjusted ln(μ)			τ			
	Effect	Heterogeneity	Bias	mean	LowerCI	UpperCI	mean	LowerCI	UpperCI	I2
Soil Organic Carbon	30	0.57	1.1	0.23	0.00	0.31	0.04	0.00	0.21	7.7
Biological	4078	1.1.E+14	0.2	0.58	0.45	0.70	0.24	0.16	0.34	63.6
Chemical	8427	3.2.E+17	0.1	0.46	0.38	0.53	0.21	0.16	0.27	54.9
Physical	2.1	0.68	1.7	0.20	0.00	0.47	0.06	0.00	0.32	7.8
Water regulation	8140	0.40	0.2	0.54	0.45	0.62	0.03	0.00	0.17	6.8
Nutrient leaching & runoff	101	11.84	0.3	0.51	0.32	0.66	0.17	0.00	0.37	39.3
Soil erosion	0.64	∞	17.3	0.06	-0.74	0.84	1.02	0.67	1.47	98.5

	RMA			Boot 95% CI		Funnel plot	Rosenthal	Coeff-test
	ln(μ)	Q	Q p-value	LowerCI	UpperCI	bias visual estimation	fail safe number	p-value
Soil Organic Carbon	0.26	40.11	0.96	0.21	0.31	yes	914	<0.001
Biological	0.47	122.90	< .0001	0.41	0.52	yes	611	0.216
Chemical	0.32	202.96	< .0001	0.28	0.36	no	2830	0.007
Physical	0.31	9.11	0.96	0.16	0.46	~yes	58	0.126
Water regulation	0.55	24.34	0.23	0.47	0.62	no	590	0.002
Nutrient leaching & runoff	0.66	29.29	0.03	0.57	0.76	yes	127	0.077
Soil erosion	1.04	2189.61	< .0001	1.00	1.08	no	90	0.010
